# Knowledge Transfer Between Artificial Intelligence Systems

**DOI:** 10.3389/fnbot.2018.00049

**Published:** 2018-08-13

**Authors:** Ivan Y. Tyukin, Alexander N. Gorban, Konstantin I. Sofeykov, Ilya Romanenko

**Affiliations:** ^1^Department of Mathematics, University of Leicestger, Leicester, United Kingdom; ^2^Laboratory of Advanced Methods for High-Dimensional Data Analysis, Lobachevsky State University of Nizhny Novgorod, Nizhny Novgorod, Russia; ^3^Imaging and Vision Group, ARM Holdings, Loughborough, United Kingdom; ^4^Spectral Edge Ltd, Cambridge, United Kingdom

**Keywords:** stochastic separation theorems, concentration of measure, knowledge transfer in artificial intelligence systems, error correction, supervised learning, neural networks

## Abstract

We consider the fundamental question: how a legacy “student” Artificial Intelligent (AI) system could learn from a legacy “teacher” AI system or a human expert without re-training and, most importantly, without requiring significant computational resources. Here “learning” is broadly understood as an ability of one system to mimic responses of the other to an incoming stimulation and vice-versa. We call such learning an Artificial Intelligence knowledge transfer. We show that if internal variables of the “student” Artificial Intelligent system have the structure of an *n*-dimensional topological vector space and *n* is sufficiently high then, with probability close to one, the required knowledge transfer can be implemented by simple cascades of linear functionals. In particular, for *n* sufficiently large, with probability close to one, the “student” system can successfully and non-iteratively learn *k* ≪ *n* new examples from the “teacher” (or correct the same number of mistakes) at the cost of two additional inner products. The concept is illustrated with an example of knowledge transfer from one pre-trained convolutional neural network to another.

## 1. Introduction

Explosive development of neuroinformatics and Artificial Intelligence (AI) in recent years gives rise to new fundamental scientific and societal challenges. Developing technologies, professions, vocations, and corresponding educational environments for sustained generation of evergrowing number of AI Systems is currently recognized as amongst the most crucial of these (Hall and Pesenti, 2017). Nurturing and growing of relevant human expertise is considered as a way to address the challenge. The next step, however, is to develop technologies whereby one or several AI systems produce a training environment for the other leading to fully automated passage of knowledge and experience between otherwise independent AI agents.

Knowledge transfer between Artificial Intelligent systems has been the subject of extensive discussion in the literature for more than two decades (Gilev et al., 1991; Jacobs et al., 1991; Pratt, 1992; Schultz and Rivest, 2000; Buchtala and Sick, 2007) (see also a comprehensive review Pan and Yang, 2010). Several technical ideas to achieve AI knowledge transfer have been explored to date. Using or salvaging, parts of the “teacher” AI system in the “student” AI followed by re-training of the “student” has been proposed and extensively tested in Yosinski et al. (2014) and Chen et al. (2015). Alternatives to AI salvaging include model compression (Bucila et al., 2006), knowledge *distillation* (Hinton et al., 2015), and *privileged information* (Vapnik and Izmailov, 2017). These approaches demonstrated substantial success in improving generalization capabilities of AIs as well as in reducing computational overheads (Iandola et al., 2016), in cases of knowledge transfer from larger AI to the smaller one. Notwithstanding, however, which of the above strategies is followed, their computational implementation, even for the case of transferring or learning just a handful of new examples, often requires either significant resources including access to large training sets and power needed for training, or availability of privileged information that may not necessarily be available to end-users. This contrasts sharply with natural intelligence too as recent empirical evidence reveals that single neurons in human brain are capable of rapid learning of new stimuli (Ison et al., 2015). Thus new frameworks and approaches are needed.

In this contribution we provide new framework for automated, fast, and non-destructive process of knowledge spreading across AI systems of varying architectures. In this framework, knowledge transfer is accomplished by means of Knowledge Transfer Units comprising of mere linear functionals and/or their small cascades. Main mathematical ideas are rooted in measure concentration (Gibbs, 1902; Lévy, 1951; Gromov, 1999, 2003; Gorban, 2007) and stochastic separation theorems (Gorban and Tyukin, 2017, 2018) revealing peculiar properties of random sets in high dimensions. We generalize some of the latter results here and show how these generalizations can be employed to build simple one-shot Knowledge Transfer algorithms between heterogeneous AI systems whose state may be represented by elements of linear vector space of sufficiently high dimension. Once knowledge has been transferred from one AI to another, the approach also allows to “unlearn” new knowledge without the need to store a complete copy of the “student” AI is created prior to learning. We expect that the proposed framework may pave way for fully functional new phenomenon—Nursery of AI systems in which AIs quickly learn from each other whilst keeping their pre-existing skills largely intact.

The paper is organized as follows. In section 2 we introduce a general framework for computationally efficient non-iterative AI Knowledge Transfer and present two algorithms for transferring knowledge between a pair of AI systems in which one operates as a teacher and the other functions as a student. These results are based on Stochastic Separation Theorems (Gorban and Tyukin, 2017) of which the relevant versions are provided here as mathematical background justifying the approach. Section 3 illustrates the approach with examples, and section 4 concludes the paper.

## 2. Non-iterative AI knowledge transfer framework

### 2.1. General setup

Consider two AI systems, a student AI, denoted as AI_*s*_, and a teacher AI, denoted as AI_*t*_. These legacy AI systems process some *input* signals, produce *internal* representations of the input and return some *outputs*. We further assume that some *relevant* information about the input, internal signals, and outputs of AI_*s*_ can be combined into a common object, ***x***, representing, but not necessarily defining, the *state* of AI_*s*_. The objects ***x*** are assumed to be elements of ℝ^*n*^.

Over a period of activity system AI_*s*_ generates a set S of objects ***x***. Exact composition of the set S could depend on a task at hand. For example, if AI_*s*_ is an image classifier, we may be interested only in a particular subset of AI_*s*_ input-output data related to images of a certain known class. Relevant inputs and outputs of AI_*s*_ corresponding to objects in S are then evaluated by the teacher, AI_*t*_. If AI_*s*_ outputs differ to that of AI_*t*_ for the same input then an error is registered in the system. Objects x∈S associated with errors are combined into the set Y. The procedure gives rise to two disjoint sets:

$$M=S\backslash Y,\;M=\left\{{\text{x}}_{1},\ldots ,{\text{x}}_{M}\right\}$$

and

$$Y=\left\{{\text{x}}_{M+1},\ldots ,{\text{x}}_{M+k}\right\}.$$

**Figure 1:**
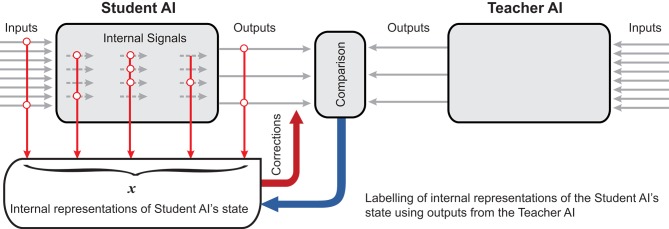
AI Knowledge transfer diagram. *AI*_*s*_ produces a set of its state representations, S. The representations are labeled by *AI*_*t*_ into the set of correct responses, M, and the set of errors, Y. The student system, *AI*_*s*_, is then augmented by an additional “corrector” eliminating these errors.

A diagram schematically representing the process is shown in Figure 1. The knowledge transfer task is to “teach” AI_*s*_ so that with

- AI_*s*_ does not make such errors
- existing competencies of AI_*s*_ on the set of inputs corresponding to internal states x∈M are retained, and
- knowledge transfer from AI_*t*_ to AI_*s*_ is reversible in the sense that AI_*s*_ can “unlearn” new knowledge by modifying just a fraction of its parameters, if required.

Before proceeding with a proposed solution to the above AI Knowledge Transfer problem, understanding basic yet fundamental properties of the sets Y and M is needed. These properties are summarized and illustrated with Theorems 1, 2, and 3 below.

### 2.2. Stochastic separation theorems for non-iterative AI knowledge transfer

Let the set

$$M=\left\{{\text{x}}_{1},\ldots ,{\text{x}}_{M}\right\}$$

be an i.i.d. sample from a distribution in ℝ^*n*^. Pick another set

$$Y=\left\{{\text{x}}_{M+1},\ldots ,{\text{x}}_{M+k}\right\}$$

from the same distribution at random. What is the probability that there is a linear functional separating Y from M, and, most importantly, if there is a computationally simple and efficient way to determine these?

Below we provide three *k*-tuple separation theorems: for an equidistribution in the unit ball *B*_*n*_(1) (Theorems 1 and 2) and for a product probability measure with bounded support (Theorem 3). These two special cases cover or, indeed, approximate a broad range of practically relevant situations including e.g., Gaussian distributions (reduce asymptotically to the equidistribution in *B*_*n*_(1) for *n* large enough) and data vectors in which each attribute is a numerical and independent random variable. The computational complexity for determining the separating functionals, as specified by the theorems and their proofs, can be remarkably low. If no pre-processing is involved, then deriving the functionals stemming from Theorems 1 and 2 requires merely k=|Y| vector additions and, possibly, an approximate solution of a constrained optimization problem in two dimensions. For large data sets, this is a significant advantage over support vector machines whose worst-case computational complexity is *O*((*k*+*M*)^3^) (Bordes et al., 2005; Chapelle, 2007).

Consider the case when the underlying probability distribution is an equidistribution in the unit ball *B*_*n*_(1), and suppose that $M=\left\{{\text{x}}_{1},\ldots ,{\text{x}}_{M}\right\}$ and $Y=\left\{{\text{x}}_{M+1},\ldots ,{\text{x}}_{M+k}\right\}$ are i.i.d. samples from this distribution. We are interested in determining the probability $P_{1}\left(M,Y\right)$ that there exists a linear functional separating M and Y. An estimate of this probability is provided in the following theorem.

**Theorem 1**. *Let* $ℳ=\{x_{1},\ldots ,x_{M}\}\;and\;Y=\{x_{M+1},\ldots ,x_{M+k}\}$ *be i.i.d. samples from the equidisribution in B_n_*(1). *Then*

$$\begin{array}{l} P_{1}(ℳ,Y)\geq \underset{\delta ,\varepsilon }{\max}1-k{\left(1-\varepsilon \right)}^{n}-\frac{(k-1)k}{2}{\left(1-\delta^{2}\right)}^{\frac{n}{2}}\\ \;-\frac{M}{2}\Delta {\left(\varepsilon ,\delta ,k\right)}^{\frac{n}{2}} \end{array}$$

(1) $$\begin{array}{l} \Delta (\varepsilon ,\delta ,k)=1-\frac{1}{k}{\left[\frac{{\left(1-\varepsilon \right)}^{2}-\frac{k-1}{1-\varepsilon }\delta }{\sqrt{1+\frac{k-1}{1-\varepsilon }\delta }}\right]}^{2} \end{array}$$

$$\begin{array}{l} \text{ Subject to}:\\ \;\delta ,\varepsilon \in (0,1)\\ \;(k-1)\delta \leq {\left(1-\varepsilon \right)}^{3}. \end{array}$$

*If the pair* (δ, ε) *is a solution of the nonlinear optimization program in (1) then the corresponding separating hyperplane is:*

$$\ell_{0}(x)=0,\;\ell_{0}(x)=\langle \frac{\bar{x}}{\left\|\bar{x}\right\|},x\rangle -\frac{1}{\sqrt{k}}\frac{{\left(1-\varepsilon \right)}^{2}-\frac{k-1}{1-\varepsilon }\delta }{\sqrt{1+\frac{k-1}{1-\varepsilon }\delta }},$$

$$\bar{x}=\frac{1}{k}\sum_{i=1}^{k}x_{M+i}.$$

The proof of the theorem is provided in the Appendix.

**Figure 2:**
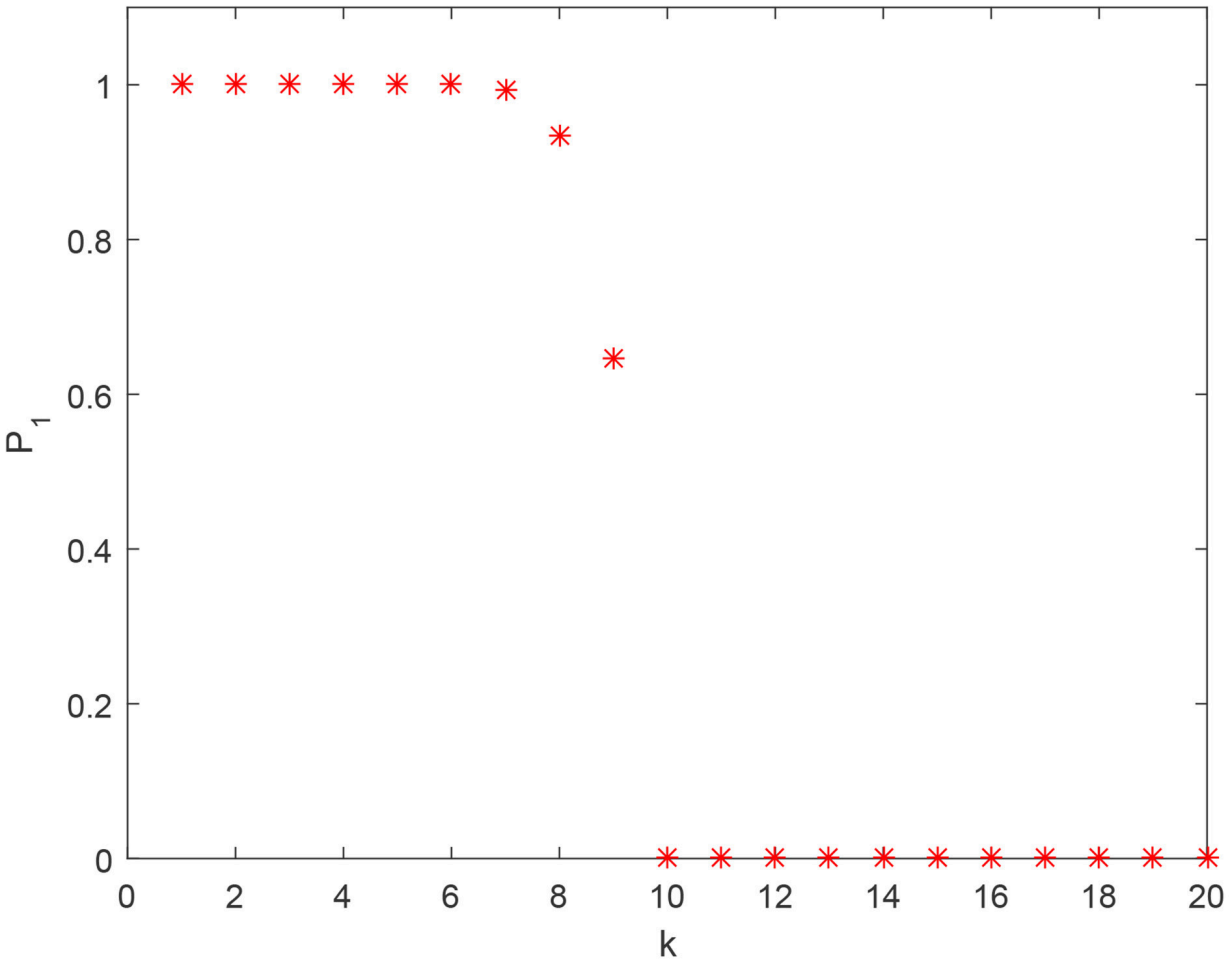
Estimate (1) of $P_{1}\left(M,Y\right)$ as a function of *k* for *n* = 2, 000 and *M* = 10^5^.

Figure 2 shows how estimate (1) of the probability $P_{1}\left(M,Y\right)$ behaves, as a function of |Y| for fixed *M* and *n*. As one can see from this figure, when *k* exceeds some critical value (*k* = 9 in this specific case), the lower bound estimate (1) of the probability $P_{1}\left(M,Y\right)$ drops. This is not surprising since the bound (1) is (A) based on conservative estimates, and (B) these estimates are derived for just one class of separating hyperplanes ℓ_0_(***x***). Furthermore, no prior pre-processing and/or clustering was assumed for the Y. An alternative estimate that allows us to account for possible clustering in the set Y is presented in Theorem 2.

**Theorem 2**. *Let* $M=\left\{{\text{x}}_{1},\ldots ,{\text{x}}_{M}\right\}$ *and* $Y=\left\{{\text{x}}_{M+1},\ldots ,{\text{x}}_{M+k}\right\}$ *be i.i.d. samples from the equidisribution in B_n_*(1). *Let* $Y_{c}=\left\{{\text{x}}_{M+r_{1}},\ldots ,{\text{x}}_{M+r_{m}}\right\}$ *be a subset of m elements from* Y *such that*

(2) $$\begin{array}{l} \beta_{2}(m-1)\leq \;\sum_{r_{j},\;r_{j}\;\neq \;r_{i}}\;\;\langle x_{M+r_{i}},x_{M+r_{j}}\rangle \;\leq \;\beta_{1}(m-1)for\;all\;i=1,\ldots ,m. \end{array}$$

Then

$$P_{1}(ℳ,Y_{c})\geq \max_{\varepsilon }{\left(1-{\left(1-\varepsilon \right)}^{n}\right)}^{k}{\left(1-\frac{\Delta {\left(\varepsilon ,m\right)}^{\frac{n}{2}}}{2}\right)}^{M}$$

(3) $$\Delta (\varepsilon ,m)=1-\frac{1}{m}{\left(\frac{{\left(1-\varepsilon \right)}^{2}+\beta_{2}(m-1)}{\sqrt{1+(m-1)\beta_{1}}}\right)}^{2}$$

$$\begin{array}{l} \text{ Subject to}:\\ \;{\left(1-\varepsilon \right)}^{2}+\beta_{2}(m-1)>0\\ \;1+(m-1)\beta_{1}>0. \end{array}$$

*If the pair* (δ, ε) *is a solution of the nonlinear optimization program in (3) then the corresponding separating hyperplane is:*

$$\ell_{0}(x)=0,\;\ell_{0}(x)=\langle \frac{\bar{y}}{\left\|\bar{y}\right\|},x\rangle -\frac{1}{\sqrt{m}}\left(\frac{{\left(1-\varepsilon \right)}^{2}+\beta_{2}(m-1)}{\sqrt{1+(m-1)\beta_{1}}}\right),$$

$$\bar{y}=\frac{1}{m}\sum_{i=1}^{m}x_{M+r_{i}}.$$

The proof of the theorem is provided in Appendix.

**Figure 3:**
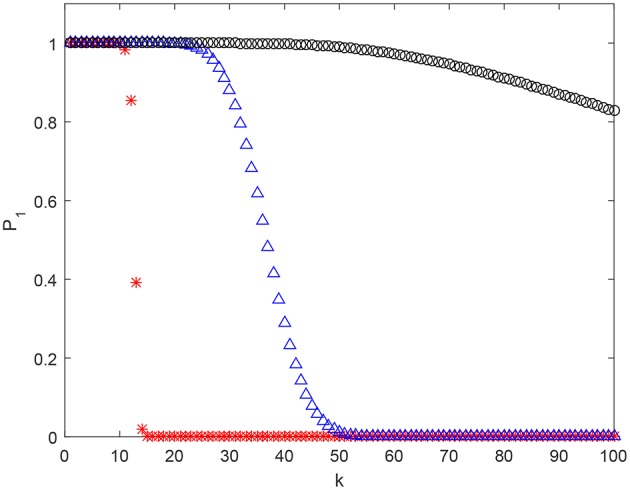
Estimate (2) of $P_{1}\left(M,Y\right)$ as a function of *k* for *n* = 2, 000 and *M* = 10^5^. Red stars correspond to β_1_ = 0.5, β_2_ = 0. Blue triangles stand for β_1_ = 0.5, β_2_ = 0.05, and black circles stand for β_1_ = 0.5, β_2_ = 0.07.

Examples of estimates (3) for various parameter settings are shown in Figure 3. As one can see, in absence of pair-wise strictly positive correlation assumption, β_2_ = 0, the estimate's behavior, as a function of *k*, is similar to that of (1). However, presence of moderate pair-wise positive correlation results in significant boosts to the values of $P_{1}$.

Remark 1. Estimates (1), (3) for the probability $P_{1}\left(M,Y\right)$ that follow from Theorems 1, 2 assume that the underlying probability distribution is an equidistribution in *B*_*n*_(1). They can, however, be generalized to equidistributions in ellipsoids and Gaussian distributions (cf. Gorban et al., 2016a,b). Tighter probability bounds could also be derived if the upper-bound estimates of the volumes of the corresponding spherical caps in the proofs of Theorems 1, 2 are replaced with their exact values (see e.g., Li, 2011).

Remark 2. Note that not only Theorems 1, 2 provide estimates from below of the probability that two random i.i.d. drawn samples from *B*_*n*_(1) are linearly separable, but also they explicitly present the separating hyperplanes. The latter hyperplanes are similar to Fisher linear discriminants in that the discriminating direction (normal to the hyperplane) is the difference between the centroids.

Whilst having explicit separation functionals as well as thresholds is an obvious advantage from practical view point, the estimates that are associated with such functionals do not account for more flexible alternatives. In what follows we present a generalization of the above results that accounts for such a possibility as well as extends applicability of the approach to samples from product distributions. The results are provided in Theorem 3.

**Theorem 3**. *Consider the linear space E* = span{***x**_j_* − ***x***_*M*+1_ | *j* = *M* + 2, …, *M* + *k*}, *let the cardinality* |Y|=k *of the set* Y *be smaller than n. Consider the quotient space* ℝ^*n*^/*E. Let Q*(***x***) *be a representation of* ***x*** ∈ ℝ^*n*^ in ℝ^*n*^/*E, and let the coordinates of Q*(***x***_*i*_), *i* = 1, …, *M* + 1 *be independent random variables i.i.d. sampled from a product distribution in a unit cube with variances* σ_*j*_ > σ_0_ > 0, 1 ≤ *j* ≤ *n* − *k* + 1. *Then for*

$$M\leq \frac{\vartheta }{3}\exp\left(\frac{\left(n-k+1\right)\sigma_{0}^{4}}{2}\right)-1$$

*with probability p* > 1 − ϑ *there is a linear functional separating* Y *and* M.

The proof of the theorem is provided in Appendix.

Having introduced Theorems 1–3, we are now ready to formulate our main results–algorithms for non-iterative AI Knowledge Transfer.

### 2.3. Knowledge transfer algorithms

Our first algorithm, Algorithm 1, considers cases when *Auxiliary Knowledge Transfer Units*, i.e. functional additions to existing student AI_*s*_, are single linear functionals. The second algorithm, Algorithm 2, extends Auxiliary Knowledge Transfer Units to two-layer cascades of linear functionals.

**Algorithm 1:**
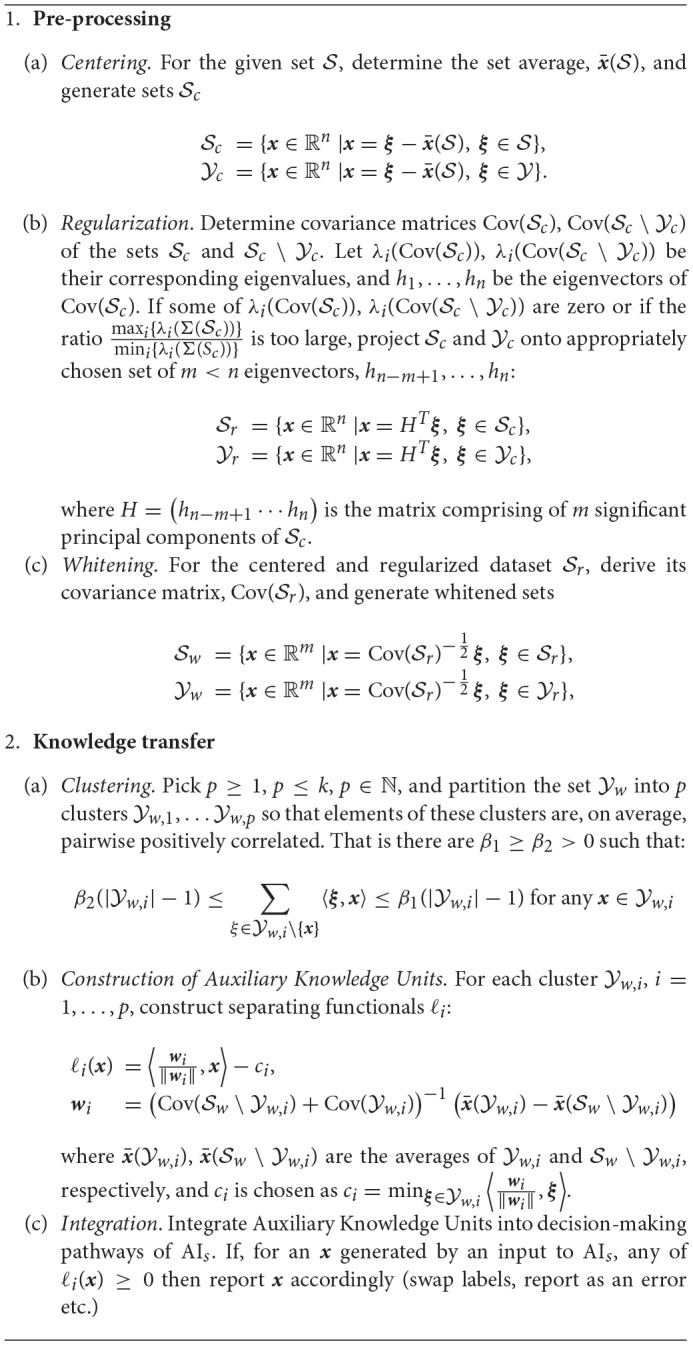
Single-functional AI Knowledge Transfer

**Algorithm 2:**
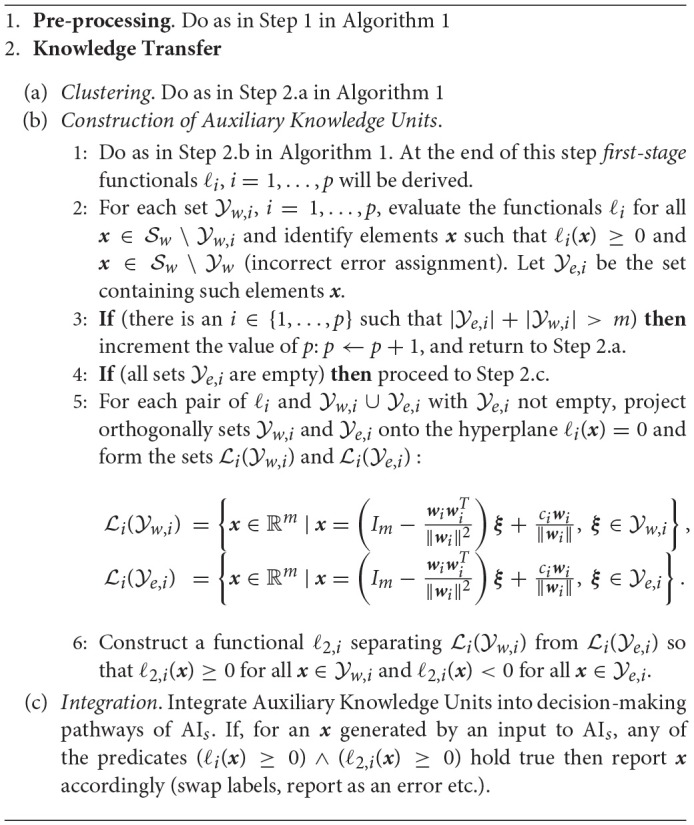
Two-functional AI Knowledge Transfer

The algorithms comprise of two general stages, pre-processing stage and knowledge transfer stage. The purpose of the pre-processing stage is to regularize and “sphere” the data. This operation brings the setup close to the one considered in statements of Theorems 1, 2. The knowledge transfer stage constructs Auxiliary Knowledge Transfer Units in a way that is very similar to the argument presented in the proofs of Theorems 1 and 2. Indeed, if $\left|Y_{w,i}|\ll |S_{w}\backslash Y_{w,i}\right|$ then the term ${\left(\text{Cov}\left(S_{w}\backslash Y_{w,i}\right)+\text{Cov}\left(Y_{w,i}\right)\right)}^{-1}$ is close to identity matrix, and the functionals ℓ_*i*_ are good approximations of (10). In this setting, one might expect that performance of the knowledge transfer stage would be also closely aligned with the corresponding estimates (1), (3).

Remark 3. Note that the regularization step in the pre-processing stage ensures that the matrix $\text{Cov}\left(S_{w}\backslash Y_{w,i}\right)+\text{Cov}\left(Y_{w,i}\right)$ is non-singular. Indeed, consider

$$\begin{array}{l} \text{Cov}(S_{w}\setminus Y_{w,i})=\frac{1}{\left|S_{w}\setminus Y_{w,i}\right|}\sum_{x\in S_{w}\setminus Y_{w,i}}(x-\bar{x}(S_{w}\setminus Y_{w,i}))\\ {\left(x-\bar{x}(S_{w}\setminus Y_{w,i})\right)}^{T}=\frac{1}{\left|S_{w}\setminus Y_{w,i}\right|}(\sum_{x\in S_{w}\setminus Y_{w}}(x-\bar{x}(S_{w}\setminus Y_{w,i}))\\ {\left(x-\bar{x}(S_{w}\setminus Y_{w,i})\right)}^{T}+\sum_{x\in Y_{w}\setminus Y_{w,i}}(x-\bar{x}(S_{w}\setminus Y_{w,i}))\\ {\left(x-\bar{x}(S_{w}\setminus Y_{w,i})\right)}^{T}). \end{array}$$

Denoting $d=\bar{\text{x}}\left(S_{w}\backslash Y_{w,i}\right)-\bar{\text{x}}\left(S_{w}\backslash Y_{w}\right)$ and rearranging the sum below as

$$\begin{array}{l} \sum_{x\in S_{w}\setminus Y_{w}}(x-\bar{x}(S_{w}\setminus Y_{w,i})){\left(x-\bar{x}\left(S_{w}\setminus Y_{w,i}\right)\right)}^{T}=\\ \sum_{x\in S_{w}\setminus Y_{w}}(x-\bar{x}(S_{w}\setminus Y_{w})+d){\left(x-\bar{x}\left(S_{w}\setminus Y_{w,i}\right)+d\right)}^{T}=\\ \sum_{x\in S_{w}\setminus Y_{w}}(x-\bar{x}(S_{w}\setminus Y_{w})){\left(x-\bar{x}(S_{w}\setminus Y_{w})\right)}^{T}+\\ 2d\sum_{x\in S_{w}\setminus Y_{w}}(x-\bar{x}(S_{w}\setminus Y_{w}){)}^{T}+|S_{w}\setminus Y_{w}|dd^{T}\\ =\sum_{x\in S_{w}\setminus Y_{w}}(x-\bar{x}(S_{w}\setminus Y_{w})){\left(x-\bar{x}(S_{w}\setminus Y_{w})\right)}^{T}\\ +|S_{w}\setminus Y_{w}|dd^{T} \end{array}$$

we obtain that $\text{Cov}\left(S_{w}\backslash Y_{w,i}\right)$ is non-singular as long as the sum $\underset{\text{x}\in S_{w}\backslash Y_{w}}{\sum }\left(\text{x}-\bar{\text{x}}\left(S_{w}\backslash Y_{w}\right)\right){\left(\text{x}-\bar{\text{x}}\left(S_{w}\backslash Y_{w}\right)\right)}^{T}$ is non-singular. The latter property, however, is guaranteed by the regularization step in Algorithm 1.

Remark 4. Clustering at Step 2.a can be achieved by classical *k*-means algorithms (Lloyd, 1982) or any other method (see e.g., Duda et al., 2000) that would group elements of $Y_{w}$ into clusters according to spatial proximity.

Remark 5. Auxiliary Knowledge Transfer Units in Step 2.b of Algorithm 1 are derived in accordance with standard Fisher linear discriminant formalism. This, however, need not be the case, and other methods, e.g., support vector machines (Vapnik, 2000), could be employed for this purpose there. It is worth mentioning, however, that support vector machines might be prone to overfitting (Han, 2014) and their training often involves iterative procedures such as sequential quadratic minimization (Platt, 1999).

Furthermore, instead of the sets $Y_{w,i}$, $S_{w}\backslash Y_{w,i}$ one could use a somewhat more aggressive division: $Y_{w,i}$ and $S_{w}\backslash Y_{w}$, respectively.

Depending on configuration of samples S and Y, Algorithm 1 may occasionally create Knowledge Transfer Units, ℓ_*i*_, that are “filtering” errors too aggressively. That is, some $\text{x}\in S_{w}\backslash Y_{w}$ may accidentally trigger non-negative response, ℓ_*i*_(***x***) ≥ 0, and as a result of this, their corresponding inputs to A_*s*_ could be ignored or mishandled. To mitigate this, one can increase the number of clusters and Knowledge Transfer Units, respectively. This will increase the probability of successful separation and hence alleviate the issue. An alternative practical strategy to limit the number of Knowledge Transfer Units, when the system is evolving in time, is to retain only most relevant ones taking into account acceptable rates of performance and size of the “relevant” set S. The link between these is provided in Theorems 1, 2. On the other hand, if increasing the number of Knowledge Transfer Units or dismissing less relevant ones is not desirable for some reason, then two-functional units could be a feasible remedy. Algorithm 2 presents a procedure for such an improved AI Knowledge Transfer.

In what follows we illustrate the approach as well as the application of the proposed Knowledge Transfer algorithms in a relevant problem of a computer vision system design for pedestrian detection in live video streams.

## 3. Example

Let *AI*_*s*_ and *AI*_*t*_ be two systems developed, e.g., for the purposes of pedestrian detection in live video streams. Technological progress in embedded systems and availability of platforms such as Nvidia Jetson TX2 made hardware deployment of such AI systems at the edge of computer vision processing pipelines feasible. These platforms, however, lack computational power that would enable to run state-of-the-art large scale object detection solutions like ResNet (He et al., 2016) in real-time. Smaller-size convolutional neural networks such as SqueezeNet (Iandola et al., 2016) could be a way to move forward. Still, however, these latter systems have hundreds of thousands trainable parameters which is typically several orders of magnitude larger than in e.g., Histograms of Oriented Gradients (HOG) based systems (Dalal and Triggs, 2005). Moreover, training these networks requires substantial computational resources and data.

In this section we illustrate application of the proposed AI Knowledge Transfer technology and demonstrate that this technology can be successfully employed to compensate for the lack of power of an edge-based device. In particular, we suggest that the edge-based system is “taught” by the state-of-the-art teacher in a non-iterative and near-real time way. Since our building blocks are linear functionals, such learning will not lead to significant computational overheads. At the same time, as we will show later, the proposed AI Knowledge Transfer will result in a major boost to the system's performance in the conditions of the experiment.

### 3.1. Definition of *AI*_*s*_ and *AI*_*t*_ and rationale

In our experiments, the teacher AI, *AI*_*t*_, was modeled by an adaptation of SqueezeNet (Iandola et al., 2016) with circa 725 K trainable parameters. The network was trained on a “teacher” dataset comprised of 554 K non-pedestrian (negatives), and 56 K pedestrian (positives) images. Positives have then been subjected to standard augmentation accounting for various geometric and color perturbations. The network was trained for 100 epochs, which took approximately 16 h on Nvidia Titan Xp to complete. The student AI, *AI*_*s*_, was modeled by a linear classifier with HOG features (Dalal and Triggs, 2005) and 2016 trainable parameters. The values of these parameters were the result of *AI*_*s*_ training on a “student” dataset, a sub-sample of the “teacher” dataset comprising of 16 K positives (55 K after augmentation) and 130 K negatives, respectively. The choice of *AI*_*s*_ and *AI*_*t*_ systems enabled us to emulate interaction between low-power edge-based AIs and their more powerful counterparts that could be deployed on a higher-spec embedded system or, possibly, on a server or in a computational cloud.

We note that for both *AI*_*t*_ and *AI*_*s*_ the set of negatives is several times larger than the set of positives. This makes the datasets somewhat unbalanced. Unbalanced datasets are not uncommon in object detection tasks. There are several reasons why such unbalanced datasets may emerge in practice. Every candidate for inclusion in the set of positives is typically subjected to thorough human inspection. This makes the process time-consuming and expensive, and as a result imposes limitations on the achievable size of the set. Negatives are generally easier to generate. Note also that the set of negatives in our experiments is essentially the set of all objects that are not pedestrians. This latter set has significantly broader spectrum of variations than the set of positives. Accounting for this larger variability without imposing any further prior assumptions or knowledge could be achieved via larger samples. This was the strategy we have adopted here.

**Figure 4:**
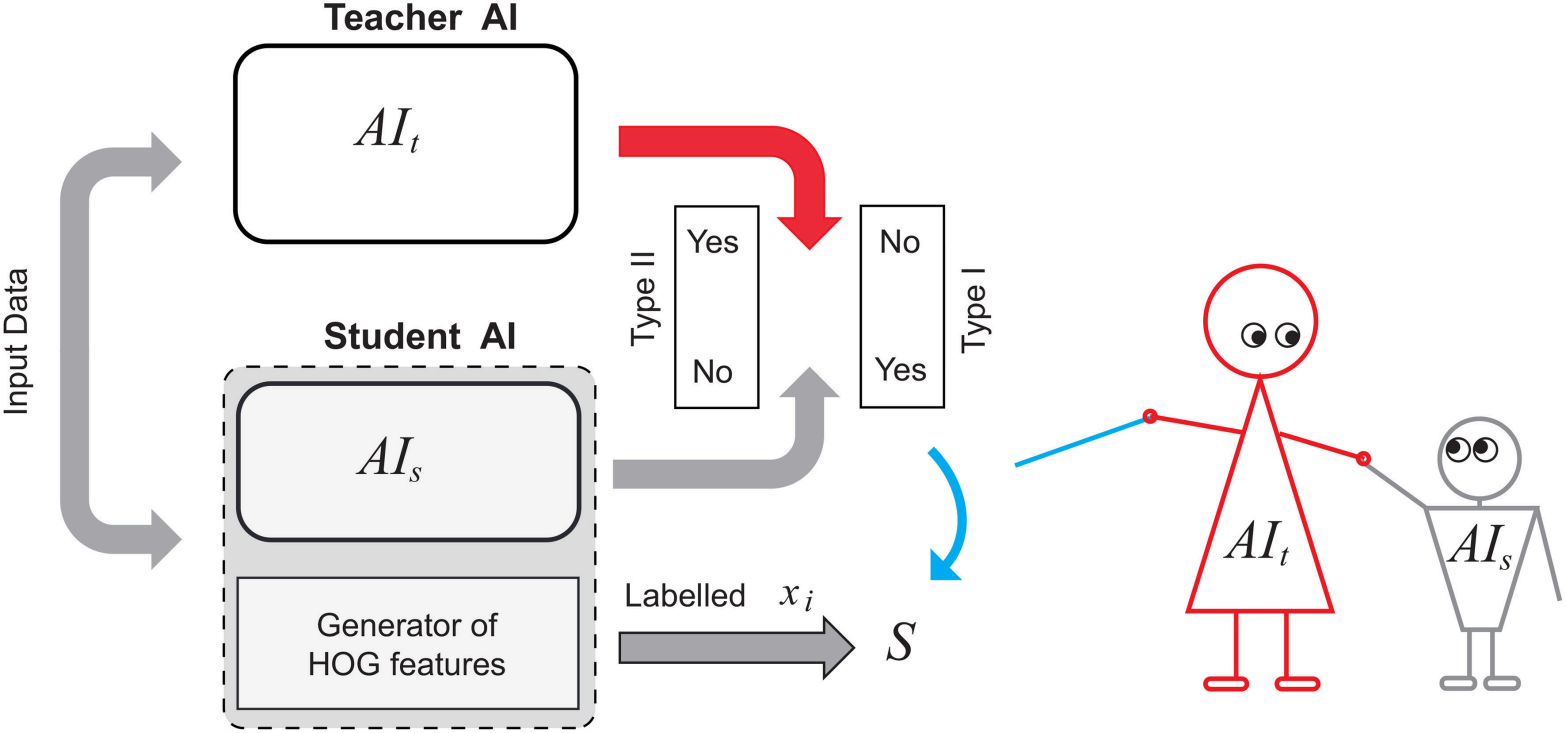
Knowledge transfer diagram between a teacher AI and a student AI augmented with HOG-based feature generator.

In order to make the experiment more realistic, we assumed that internal states of both systems are inaccessible for direct observation. To generate sets S and Y required in Algorithms 1 and 2 we augmented system *AI*_*s*_ with an external generator of HOG features of the same dimension. We assumed, however, that positives and negatives from the “student” dataset are available for the purposes of knowledge transfer. A diagram representing this setup is shown in Figure 4. A candidate image is evaluated by two systems simultaneously as well as by a HOG features generator. The latter generates 2016 dimensional vectors of HOGs and stores these vectors in the set S. If outputs of *AI*_*s*_ and *AI*_*t*_ do not match then the corresponding feature vector is added to the set Y.

### 3.2. Error types addressed

In this experiment we consider and address two types of errors: false positives (original Type I errors) and false negatives (original Type II errors). The error types were determined as follows. An error is deemed as *false positive* (for the original data) if *AI*_*s*_ reported presence of a correctly sized full-figure image of pedestrian in a given image patch whereas no such object was there. Similarly, an error is deemed as *false negative* (for the original data) if a pedestrian was present in the given image patch but *AI*_*s*_ did not report it there.

Our main focus was to replicate a deployment scenario in which *AI*_*t*_ is capable of evaluating only small image patches at once in a given processing quanta. At the same time *AI*_*s*_ is supposed to be able to process whole frame in reasonable time, but its accuracy is lower. This makes assessment of all relevant images by *AI*_*t*_ not viable computationally and, in addition, rules out automated detection of Type II errors (original false negatives) when *AI*_*s*_ is scanning the image at thousands of positions per frame. On the other hand, the number of positive responses of *AI*_*s*_ is limited by several dozens of smaller size patches per frame, which is assumed to be well within the processing capabilities of *AI*_*t*_. In view of these considerations, we therefore focused mainly on errors of Type I (original false positives). Nevertheless, in section 3.4 we discuss possible ways to handle Type II errors in the original system and provide an illustrative example of how this might be done.

**Figure 5:**
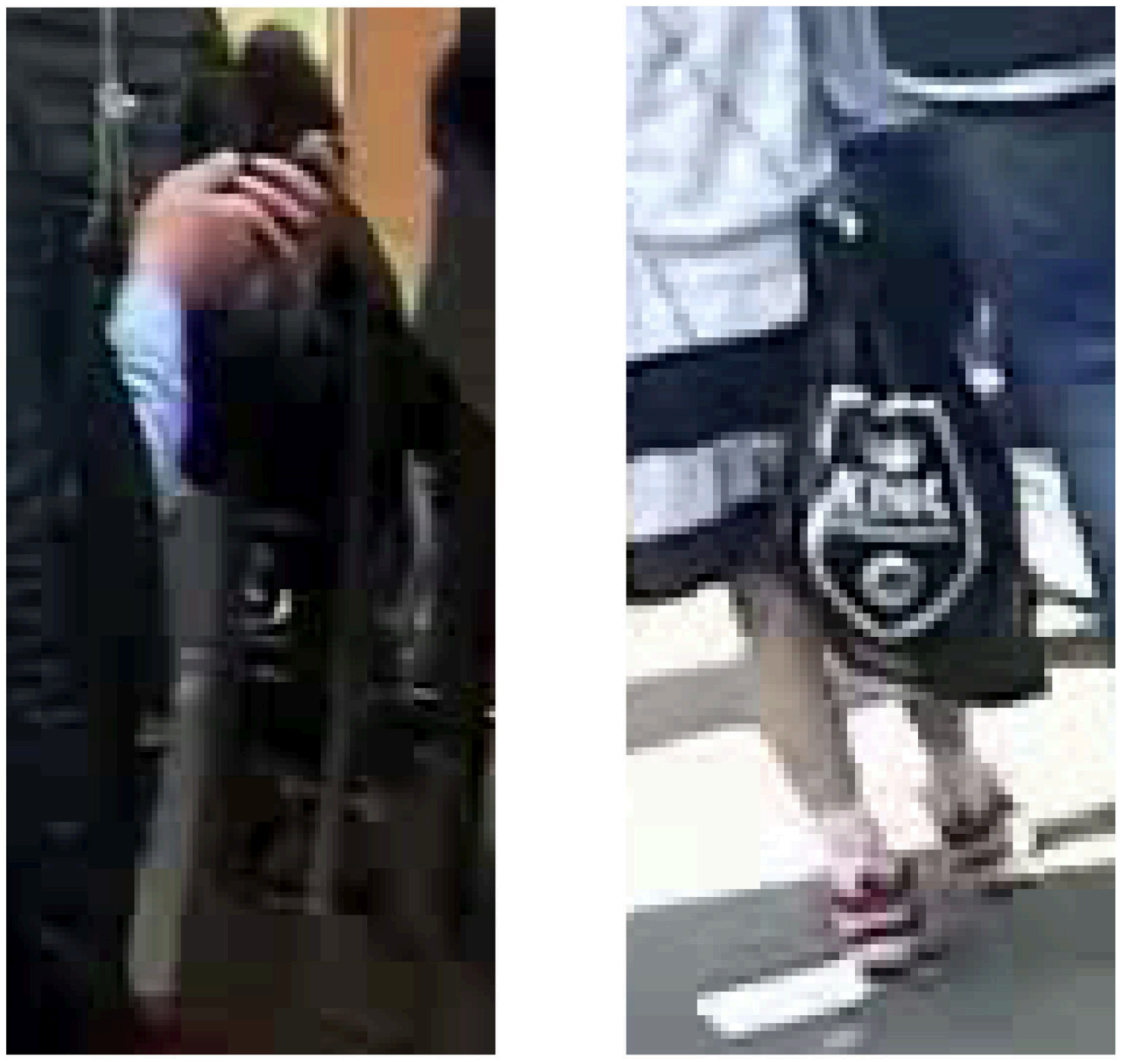
Examples of False positives generated by the teacher AI, *AI*_*t*_, for NOTTINGHAM video (Burton, 2016).

It is worthwhile to mention that output labels of the chosen teacher AI, *AI*_*t*_, do not always match ground truth labels. *AI*_*t*_ may make an occasional error too, and examples of such errors are provided in Figure 5. Regardless of these, performance of *AI*_*t*_ was markedly superior to that of *AI*_*s*_ and hence for the sake of testing the concept, these rare occasional errors of *AI*_*t*_ have been discarded in the experiments.

### 3.3. Datasets used in experiments and experimental error types

To test the approach we used NOTTINGHAM video (Burton, 2016) containing 435 frames of live footage taken with an action camera. The video, as per manual inspection, contains 4,039 full-figure images of pedestrians.

For the purposes of training and testing Knowledge Transfer Units, the video has been passed through *AI*_*s*_, and *AI*_*s*_ returned detects of pedestrian shapes. These detects were assessed by *AI*_*t*_ and labeled accordingly (see the diagram in Figure 4). At this stage, decision-making threshold in *AI*_*s*_ was varying from −0.3 to 2 to capture a reasonably large sample of false positives whose scores are near the decision boundary. This labeled set of feature vectors has been partitioned into two non-overlapping subsets: Set 1 consisting of circa 90% of true positives and 90% of false positives, and Set 2 being its complement. HOG features corresponding to original Type I errors (false positives) in Set 1 as well as all HOG features extracted from 55 K images of positives that have been used to train *AI*_*s*_ were combined into the *training set*. This training set was then used to derive Knowledge Transfer Units for *AI*_*s*_.

Sets 1 and 2 constitute different *testing sets* in our example. The first testing set (Set 1) enables us to assess how the modified *AI*_*s*_ copes with removing “seen” original Type I errors (false positives) in presence of “unseen” true positives. The second testing set (Set 2) will be used to assess generalization capabilities of the final system.

We note that labeling of false positives involved outputs of *AI*_*t*_ rather than ground truth labels. Visual inspection of *AI*_*t*_ labels revealed that they contain few dozens of false positives too. This number, however, is negligibly small as compared to the overall number of true positives (circa 2, 000) and false positives (circa 800) of student AI, *AI*_*s*_.

Finally, to quantify performance of the proposed knowledge transfer approach, it is important to distinguish between definitions of error types (Type I and Type II) for the original system and error types characterizing *performance of the Knowledge Transfer Units* themselves. The corresponding definitions are provided in Table 1. Note that true negatives (marked by star, *, in the table) do not occur in the experiments. If what follows and unless stated otherwise we shall refer to these definitions.

**Table 1:** Definition of the error types in knowledge transfer experiments.

**Response of *AI*_*t*_**	**Response of *AI*_*s*_ after**	**Error type**
	**knowledge transfer**	
Yes	Yes	True positive
	No	False negative
No	Yes	False positive
	No	True negative^*^

Results of the application of Algorithms 1, 2 as well as the analysis of their performance on the testing sets are provided below.

### 3.4. Results

We generated 10 different realizations of Sets 1 and 2. This resulted in 10 different samples of the training and testing sets. The algorithms have been applied to all these different combinations. Single run of the preprocessing step, Step 1, took, on average, 23.5 s to complete on an Apple laptop with 3.5 GHz A7 processor. After the pre-processing step only 164 principal components have been retained. This resulted in significant reduction of dimensionality of the feature vectors. In our experiments pre-processing also included normalization of the whitened vectors so that their *L*_2_ norm was always one. This brings the data onto the unit sphere which is somewhat more aligned with Theorems 1 and 2. Steps 2 in Algorithms 1, 2 took 1 and 24 ms, respectively. This is a major speed-up in comparison to complete re-training of *AI*_*s*_ (several minutes) or *AI*_*t*_ (hours). Note also that complete re-training does not offer any guarantees that the errors are going to be mitigated either.

**Figure 6:**
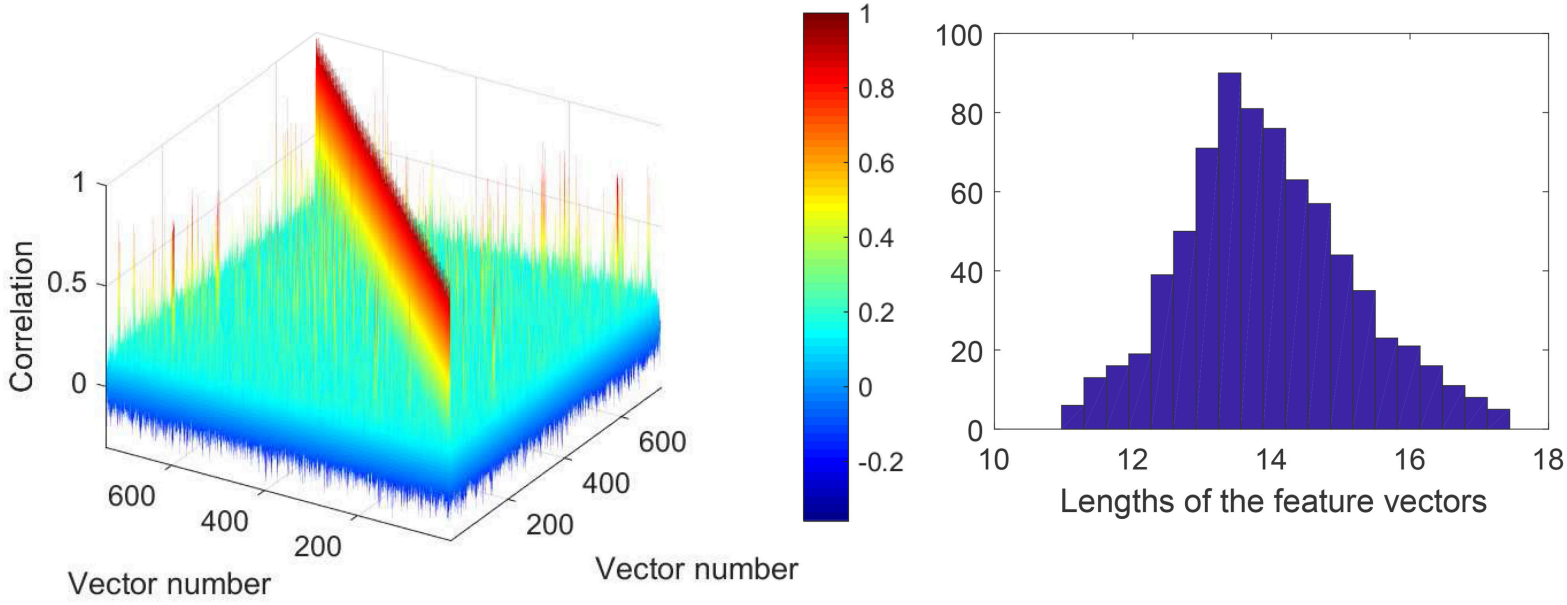
**(Left)**: Correlation diagram between elements of the set Y (elements to be learned away by *AI*_*s*_). **(Right)**: Histogram of lengths of the feature vectors in the training set after pre-processing.

**Figure 7:**
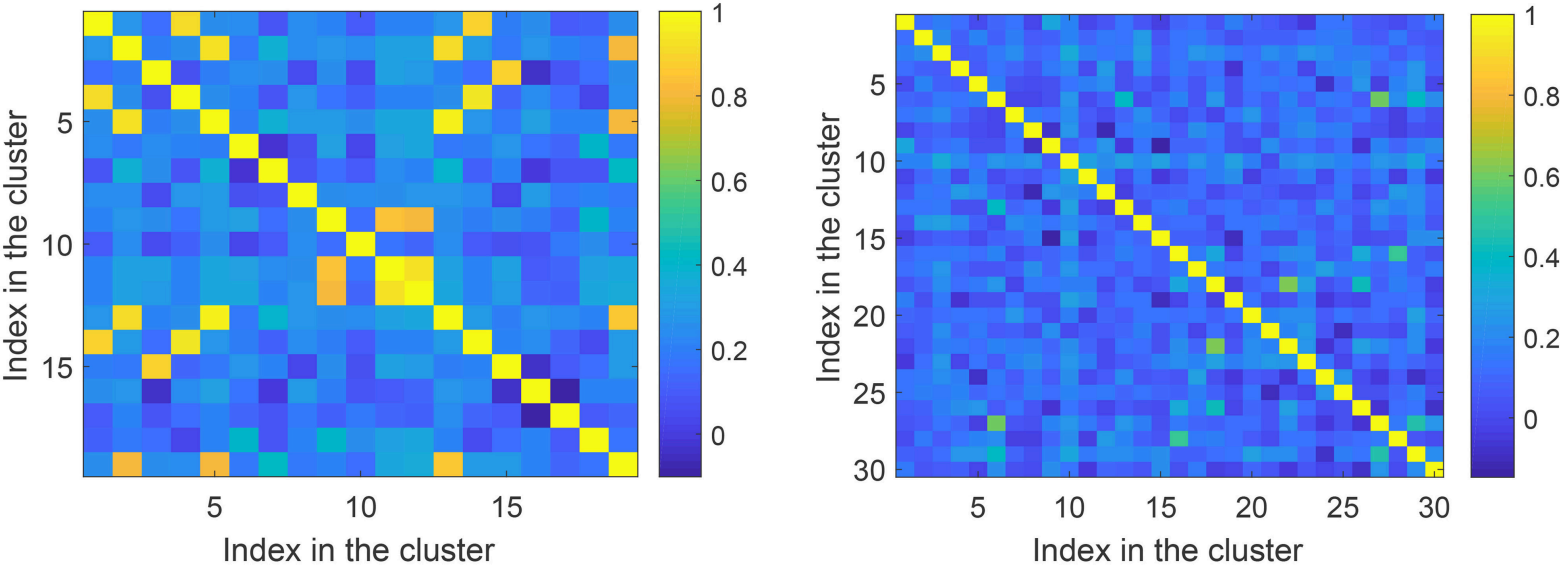
Correlations within clusters after Step 2. **Left panel** corresponds to a cluster with large threshold ≃0.76. **Right panel** corresponds to a cluster with a lower threshold ≃0.43.

Prior to running Steps 2 of the algorithms we checked if the feature vectors corresponding to errors (false positives) in the training set are correlated. This allows an informed choice of the number of clusters parameter, *p*, in Algorithms 1 and 2. A 3D color-coded visualization of correlations, i.e., 〈***x***_*i*_, ***x***_*j*_〉, between pre-processed (after Step 1) elements in the set Y is shown in Figure 6, left panel. A histogram of lengths of all vectors in the training set is shown in Figure 6, right panel. Observe that the lengths concentrate neatly around $\sqrt{164}$, as expected. According to Figure 6, elements of the set Y are mostly uncorrelated. There are, however, few correlated elements which, we expect, will be accounted for in Step 2.a of the algorithms. In absence of noticeably wide-spread correlations we set *p* = 30 which is equivalent to roughly 25 elements per cluster. Examples of correlations within clusters after Step 2 was complete are shown in Figure 7. Note that the larger is the threshold the higher is the expected correlation between elements, and the higher are the chances that such Knowledge Transfer Unit would operate successfully (see Theorems 1 and 2).

**Figure 8:**
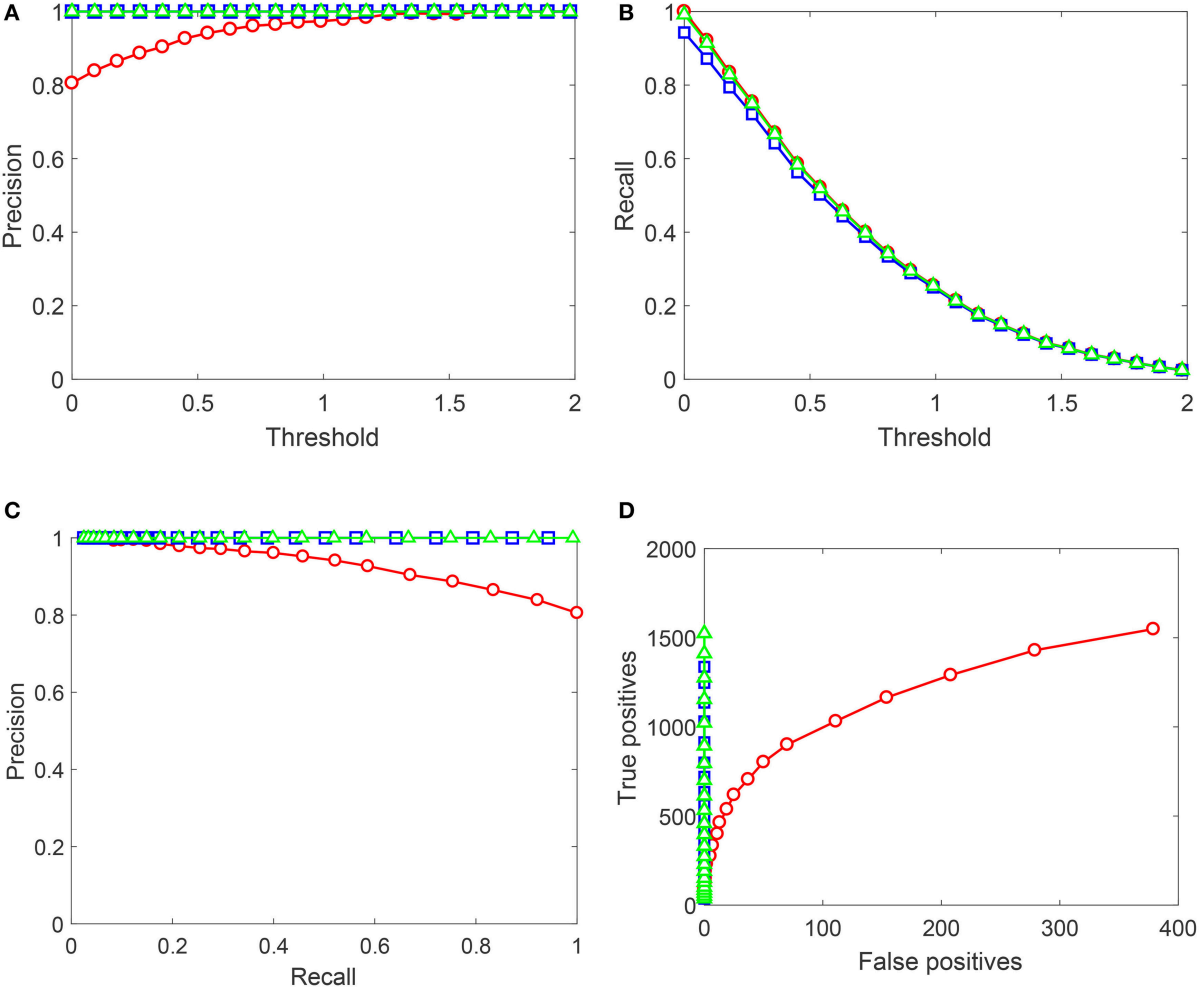
Performance of the up-trained *AI*_*s*_ on Testing set 1 (Set 1). Red circles correspond to the original *AI*_*s*_ without Knowledge Transfer Units. Blue squares correspond to *AI*_*s*_ after Algorithm 1, and green triangles illustrate application of Algorithm 2. **(A)** Shows Precision (averaged over 10 realizations of Set 1) as a function of the decision threshold in *AI*_*s*_, **(B)** shows Recall (averaged over 10 realizations of Set 1), **(C)** contains the corresponding Precision-Recall data, and **(D)** shows True positives vs. False positives for a single realization of Set 1. Lines between points are shown for the sake of visualization and do not represent any measurements or indicate achievable performance.

**Figure 9:**
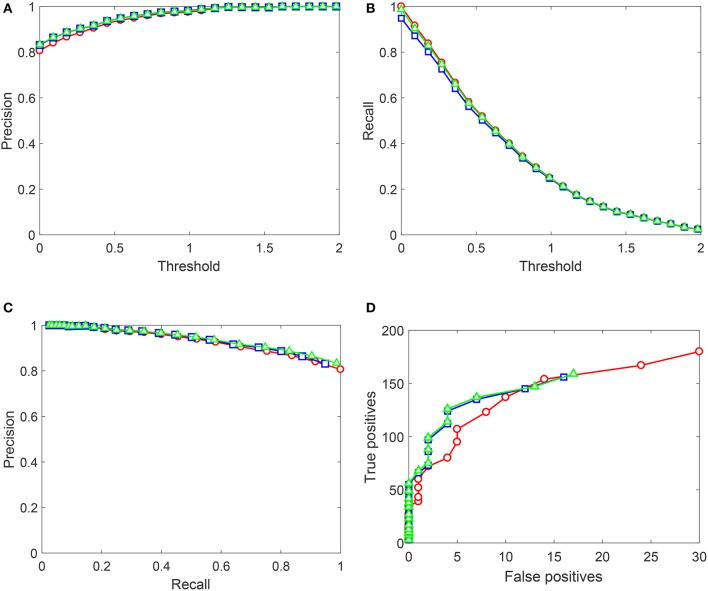
Performance of the up-trained *AI*_*s*_ on Testing set 2 (Set 2). Red circles correspond to the original *AI*_*s*_ without Knowledge Transfer Units. Blue squares correspond to *AI*_*s*_ after Algorithm 1, and green triangles illustrate application of Algorithm 2. **(A)** Shows Precision (averaged over 10 realizations of Set 2) as a function of the decision threshold in *AI*_*s*_, **(B)** shows Recall (averaged over 10 realizations of Set 2), **(C)** contains the corresponding Precision-Recall data, and **(D)** shows True positives vs. False positives for a single realization of Set 2. Lines between points are shown for the sake of visualization and do not represent any measurements or indicate achievable performance.

Performance of Algorithms 1, 2 on the Testing sets generated from NOTTINGHAM video is summarized in Figures 8, 9. In these figures we showed behavior of

$$\begin{array}{cc} \text{Precision}=\; & \frac{\text{True positives}}{\text{True positives}+\text{False positives}}\\ \text{Recall}=\; & \frac{\text{True positives}}{\text{True positives}+\text{False negatives}}, \end{array}$$

as functions of decision-making threshold in *AI*_*s*_, Precision-Recall and True positives vs. False positives charts. Red circles correspond to the original *AI*_*s*_ without Knowledge Transfer Units. Blue squares correspond to *AI*_*s*_ after Algorithm 1, and green triangles illustrate application of Algorithm 2. Note that the maximal number of false positives in Figure 8 does not exceed 400. This is due to that the threshold was now varied in the operationally feasible interval [0, 2] as opposed to [−0.3, 2] used for gather training data.

As Figure 9 shows, performance of *AI*_*s*_ on Testing set 1 (Set 1) improves drastically after the application of both algorithms. Algorithm 2 outperforms Algorithm 1 by some margin which is most noticeable from the plot in Figure 8D. Near-ideal performance in Precision-Recall space can be explained by that the training set contained full information about false positives (but not true positives). It is important to observe that Algorithm 2 did not flag nearly all “unseen” true positives.

As for results shown in Figure 9, performance patterns of *AI*_*s*_ after the application of both algorithms change. Algorithm 1 results in a minor drop in Recall figures, and Algorithm 2 recovers this drop to the baseline performance. Precision improves slightly for both algorithms, and True positives vs. False positives curves for *AI*_*s*_ with Knowledge Transfer Units dominate those of plain *AI*_*s*_. This suggests that not only the proposed Knowledge Transfer framework allows to acquire new knowledge as specified by labeled data but also has a capacity to generalize the knowledge further. The degree of such generalization will obviously depend on statistics of the data. Yet, as Figure 9 demonstrates, this is a viable possibility.

Our experiments showed how the approach could be used for filtering Type I errors in the original system. The technology, however, could be used to recover Type II errors too (false negatives in the original system), should the data be available. Several strategies might be evoked to obtain this data. The first approach is to use background subtraction to detect a moving object and pass the object through both *AI*_*s*_ and *AI*_*t*_. The second approach is to enable *AI*_*s*_ to report detects that are classified as negatives but still are reasonably close to the detection boundary. This is the strategy which we adopted here. We validated HOG features in *AI*_*s*_ corresponding to scores in the interval [−0.3, 0] with the teacher AI, *AI*_*t*_. Overall, 717 HOG vectors have been extracted by this method, of which 307 have been labeled by *AI*_*t*_ as positives, and 410 were considered as negatives. We took one of the HOG feature vectors labeled as True positive, ***x***_*v*_, and constructed (after applying the pre-processing transformation from previous experiments) several separating hyperplanes with the weights given by ***x***_*v*_/||***x***_*v*_|| and thresholds *c*_*v*_ varying in [0, 1]. Results are summarized in Table 2. As before, we observe strong concentration of measure effect: the Knowledge Transfer Unit shows extreme selectivity for sufficiently large values of *c*_*v*_. In this particular case *c*_*v*_ = 0.25 provides maximal gain at the lowest risk of expected error in future [see. e.g., the right-hand side of (1) in Theorem 1 at *k* = 1, ε = 0.75 for an estimate].

**Table 2:** Recovering Type II errors of the original *AI*_*s*_.

**Number of false negatives converted (out of 307)**	**Number of false positives remained (out of 410)**	**Threshold**
137	169	0.05
87	86	0.1
45	35	0.15
10	14	0.2
8	2	0.25
1	0	0.3

## 4. Conclusion

In this work we proposed a framework for instantaneous knowledge transfer between AI systems whose internal state used for decision-making can be described by elements of a high-dimensional vector space. The framework enables development of non-iterative algorithms for knowledge spreading between legacy AI systems with heterogeneous non-identical architectures and varying computing capabilities. Feasibility of the framework was illustrated with an example of knowledge transfer between two AI systems for automated pedestrian detection in video streams.

In the basis of the proposed knowledge transfer framework are separation theorems (Theorem 1–3) stating peculiar properties of large but finite random samples in high dimension. According to these results, *k* < *n* random i.i.d. elements can be separated form *M* ≫ *n* randomly selected elements i.i.d. sampled from the same distribution by few linear functionals, with high probability. The theorems are proved for equidistributions in a ball and in a cube. The results can be trivially generalized to equidistributions in ellipsoids and Gaussian distributions. Discussing these in detail here is the beyond the scope and vision of this work. Nevertheless, generalizations to other meaningful distributions, relaxation of the independence requirement, and a broader view on how the proposed technology could be used in multiagent AI systems is presented in technical report (Gorban et al., 2018).

## Author contributions

All authors listed have made a substantial, direct and intellectual contribution to the work, and approved it for publication.

### Conflict of interest statement

KS was employed by ARM Holding, and IR was employed by Spectral Edge Ltd. At the time of preparing the manuscript IR was employed by ARM Holding. All data used in experiments have been provided by ARM Holding. The remaining authors declare that the research was conducted in the absence of any commercial or financial relationships that could be construed as a potential conflict of interest.

## Figures and Tables

**Figure 10:**
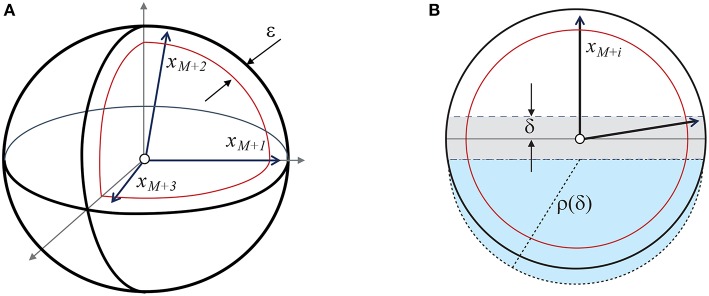
Illustration to the proof of Theorem 1. **(A)** Shows ***x***_*M*+1_, ***x***_*M*+2_, and ***x***_*M*+3_ in the set *B*_*n*_(1) \ *B*_*n*_(1−ε). **(B)** shows ***x***_*M*+*i*_ and the corresponding disc *D*_δ_(***x***_*M*+*i*_) (shaded gray area). The combined volume of the spherical caps below and above the disc is bounded from above by the volume of the *n*-ball with radius $\rho \left(\delta \right)=\sqrt{1-\delta^{2}}$. The corresponding half-ball of radius ρ(δ) is shown as the blue shaded area.

## Appendix

*Proof of Theorem 1*. Consider the set Y. The probability that a single element of Y belongs to *B*_*n*_(1) \ *B*_*n*_(1 − ε) is 1 − (1 − ε)^*n*^. Recall that if *A*_1_, …, *A*_*m*_ are arbitrary events then

(4) $$\begin{array}{l} P\left(A_{1}\&A_{2}\&\cdots \&A_{m}\right)\geq 1-\overset{m}{\underset{i\;=\;1}{\sum }}\left(1-P\left(A_{i}\right)\right). \end{array}$$

According to (4), the probability that $Y\subset B_{n}\left(1\right)\backslash B_{n}\left(1-\varepsilon \right)$ (event *E*_1_) satisfies

$$P(E_{1})\geq 1-k{\left(1-\varepsilon \right)}^{n}.$$

Pick ${\text{x}}_{M+i}\in Y$, and consider the largest equator of the ball *B*_*n*_(1) that is orthogonal to the element ***x***_*M*+*i*_. Let *D*_δ_(***x***_*M*+*i*_) denote the δ-thickening of the disc associated with this equator. Only one such disc exists, and it is uniquely defined by ***x***_*M*+*i*_ and δ (see Figure A1). Consider the following events:

$$\begin{array}{l} x_{M+2}\in D_{\delta }(x_{M+1})\text{ : event }E_{2},\\ {}[x_{M+3}\in D_{\delta }(x_{M+1})]\&[x_{M+3}\in D_{\delta }(x_{M+2})]\text{ : event }E_{3},\\ \cdots \\ {}[x_{M+k}\in D_{\delta }(x_{M+1})]\&[x_{M+k}\in \;D_{\delta }(x_{M+2})]\&\ldots \&[x_{M+k}\in \\ D_{\delta }(x_{M+k-1})]\text{ : event }E_{k}. \end{array}$$

According to (4) and Figure A1, [cf. Gorban et al., 2016b, proof of Proposition 3 and estimate (26)], it is hence clear that

$$\begin{array}{l} P(E_{2})\geq 1-{\left(1-\delta^{2}\right)}^{\frac{n}{2}},\;P(E_{3})\geq 1-2{\left(1-\delta^{2}\right)}^{\frac{n}{2}},\ldots ,\;P(E_{k})\geq \\ \;1-(k-1){\left(1-\delta^{2}\right)}^{\frac{n}{2}}. \end{array}$$

Suppose that event *E*_1_ (||***x***_*M*+*i*_|| ≥ 1−ε for all *i* = 1, …, *k*) and events *E*_2_, …, *E*_*k*_ occur. Then

$$|\cos(x_{M+i},x_{M+j})|\leq \frac{\delta }{\left(1-\varepsilon \right)}\text{ for all }i\neq j,\;i=1,\ldots ,k,$$

and

(5) $$\begin{array}{l} -\frac{\delta }{\left(1-\varepsilon \right)}\leq \langle {\text{x}}_{M+i},{\text{x}}_{M+j}\rangle \leq \frac{\delta }{\left(1-\varepsilon \right)}\;\text{for all}i\neq j,\;i=1,\ldots ,k. \end{array}$$

Consider the vector

$$\bar{x}=\frac{1}{k}\sum_{i\;=\;1}^{k}x_{M+i}.$$

Equation (5) implies that

(6) $$\begin{array}{l} \frac{1}{k}\left({\left(1-\varepsilon \right)}^{2}-\frac{k-1}{1-\varepsilon }\delta \right)\leq \langle \bar{x},x_{M+i}\rangle \leq \frac{1}{k}\left(1+\frac{k-1}{1-\varepsilon }\delta \right)\\ \text{ for all }i=1,\ldots ,k \end{array}$$

and, consequently,

(7) $$\begin{array}{l} ||\bar{\text{x}}|{|}^{2}=\langle \bar{\text{x}},\bar{\text{x}}\rangle =\frac{1}{k}\overset{k}{\underset{i\;=\;1}{\sum }}\langle \bar{\text{x}},{\text{x}}_{M+i}\rangle \leq \frac{1}{k}\left(1+\frac{k-1}{1-\varepsilon }\delta \right). \end{array}$$

Finally, consider

(8) $$\begin{array}{l} \ell_{0}\left(\text{x}\right)=\langle \frac{\bar{\text{x}}}{\left||\bar{\text{x}}|\right|},\text{x}\rangle -\frac{1}{\sqrt{k}}\frac{{\left(1-\varepsilon \right)}^{2}-\frac{k-1}{1-\varepsilon }\delta }{\sqrt{1+\frac{k-1}{1-\varepsilon }\delta }}. \end{array}$$

It is clear that if ||***x***_*M*+*i*_|| ≥ 1−ε and (5) hold then (6), (7) assure that ℓ_0_(***x***_*M*+*i*_) ≥ 0 for all ${\text{x}}_{M+i}\in Y$. The hyperplane ℓ_0_(***x***) = 0 partitions the unit ball *B*_*n*_(1) into the union of two disjoint sets: the spherical cap C

(9) $$\begin{array}{l} C=\left\{\text{x}\in B_{n}\left(1\right)\;|\ell_{0}\left(\text{x}\right)\geq 0\right\} \end{array}$$

and its complement in *B*_*n*_(1), $B_{n}\left(1\right)\backslash C$. The volume V of the cap C can be estimated from above as

$$V(C)\leq V(B_{n}(1))\frac{\Delta {\left(\varepsilon ,\delta ,k\right)}^{\frac{n}{2}}}{2},$$

$$\Delta (\varepsilon ,\delta ,k)=1-{\left[\frac{1}{\sqrt{k}}\frac{{\left(1-\varepsilon \right)}^{2}-\frac{k-1}{1-\varepsilon }\delta }{\sqrt{1+\frac{k-1}{1-\varepsilon }\delta }}\right]}^{2}.$$

Hence the probability that ℓ_0_(***x***_*i*_) < 0 for all ${\text{x}}_{i}\in M$ (event *E*_*k*+1_) can be estimated from below as

$$P(E_{k+1})\geq 1-\frac{M}{2}\Delta {\left(\varepsilon ,\delta ,k\right)}^{\frac{n}{2}},$$

and

$$\begin{array}{l} P(E_{1}E_{2}\cdots E_{k}E_{k+1})\geq 1-k{\left(1-\varepsilon \right)}^{n}-\frac{(k-1)k}{2}{\left(1-\delta^{2}\right)}^{\frac{n}{2}}\\ \;-\frac{M}{2}\Delta {\left(\varepsilon ,\delta ,k\right)}^{\frac{n}{2}}. \end{array}$$

This is a lower bound for the probability that the M can be separated from Y by the hyperplanes ℓ_0_(***x***) = 0. Given that this estimate holds for all feasible values of ε, δ, statement (1) follows. □

*Proof of Theorem 2*. Consider the set Y. Observe that, since elements ***x***_*i*_ are drawn independently from *B*_*n*_(1), ||***x***_*M*+*i*_|| ≥ 1−ε, ε ∈ (0, 1) for all *i* = 1, …, *k*, with probability *p* = (1 − (1 − ε)^*n*^)*k*. Consider now the vector $\bar{\text{y}}$

$$\bar{y}=\frac{1}{m}\sum_{i=1}^{m}x_{M+r_{i}},$$

and evaluate the following inner products

$$\begin{array}{l} \langle \frac{\bar{y}}{\left\|\bar{y}\right\|},x_{M+r_{i}}\rangle =\frac{1}{m\|\bar{y}\|}\left(\langle x_{M+r_{i}},x_{M+r_{i}}\rangle +\sum_{r_{j},\;j\neq i}\langle x_{M+r_{i}},x_{M+r_{j}}\rangle \right),\\ \;i=1,\ldots ,m. \end{array}$$

According to assumption (2),

$$\langle \frac{\bar{y}}{\left\|\bar{y}\right\|},x_{M+r_{i}}\rangle \geq \frac{1}{m\|\bar{y}\|}\left({\left(1-\varepsilon \right)}^{2}+\beta_{2}(m-1)\right)$$

and, respectively,

$$\frac{1}{m}\left(1+(m-1)\beta_{1}\right)\geq \langle \bar{y},\bar{y}\rangle \geq \frac{1}{m}\left({\left(1-\varepsilon \right)}^{2}+\beta_{2}(m-1)\right)$$

Let ${\left(1-\varepsilon \right)}^{2}+\beta_{2}\left(m-1\right)>0$ and ${\left(1-\varepsilon \right)}^{2}+\beta_{1}\left(m-1\right)>0$. Consider

(10) $$\begin{array}{l} \ell_{0}\left(\text{x}\right)=\langle \frac{\bar{\text{y}}}{\left||\bar{\text{y}}|\right|},\text{x}\rangle -\frac{1}{\sqrt{m}}\left(\frac{{\left(1-\varepsilon \right)}^{2}+\beta_{2}\left(m-1\right)}{\sqrt{1+\left(m-1\right)\beta_{1}}}\right). \end{array}$$

It is clear that ℓ_0_(***x***_*M*+_*r*__*i*__) ≥ 0 for all *i* = 1, …, *m* by the way the functional is constructed. The hyperplane ℓ_0_(***x***) = 0 partitions the ball *B*_*n*_(1) into two sets: the set C defined as in (9) and its complement, $B_{n}\left(1\right)\backslash C$. The volume V of the set C is bounded from above as

$$V(C)\leq V(B_{n}(1))\frac{\Delta {\left(\varepsilon ,m\right)}^{\frac{n}{2}}}{2}$$

where

$$\Delta (\varepsilon ,m)=1-\frac{1}{m}{\left(\frac{{\left(1-\varepsilon \right)}^{2}+\beta_{2}(m-1)}{\sqrt{1+\beta_{1}(m-1)}}\right)}^{2}.$$

Estimate (3) now follows. □

*Proof of Theorem 3*. Observe that, in the quotient space ℝ^*n*^/*E*, elements of the set

$$\begin{array}{l} Y=\{x_{M+1},x_{M+1}+(x_{M+2}-x_{M+1}),\ldots ,x_{M+1}\\ \;+(x_{M+k}-x_{M+1})\} \end{array}$$

are vectors whose coordinates coincide with that of the quotient representation of ***x***_*M*+1_. This means that the quotient representation of Y consists of a single element, *Q*(***x***_*M*+1_). Furthermore, dimension of ℝ^*n*^/*E* is *n* − *k* + 1. Let $R_{0}^{2}=\overset{n-k+1}{\underset{i=1}{\sum }}\sigma_{i}^{2}$ and $\bar{Q}\left(\text{x}\right)=𝔼\left(Q\left(\text{x}\right)\right)$. According to Theorem 2 and Corollary 2 from (Gorban and Tyukin, 2017), for ϑ ∈ (0, 1) and *M* satisfying

$$\begin{array}{l} M\leq \frac{\vartheta }{3}\exp\left(\frac{\left(n-k+1\right)\sigma_{0}^{4}}{2}\right)-1, \end{array}$$

with probability *p* > 1−ϑ the following inequalities hold:

$$\begin{array}{l} \frac{1}{2}\leq \frac{||Q\left({\text{x}}_{j}\right)-\bar{Q}\left(\text{x}\right)|{|}^{2}}{R_{0}^{2}}\leq \frac{3}{2},\;\langle \frac{Q\left({\text{x}}_{i}\right)-\bar{Q}\left(\text{x}\right)}{R_{0}},\frac{Q\left({\text{x}}_{M+1}\right)-\bar{Q}\left(\text{x}\right)}{\left||Q\left({\text{x}}_{M+1}\right)-\bar{Q}\left(\text{x}\right)|\right|}\rangle \\ <\frac{1}{\sqrt{2}} \end{array}$$

for all *i, j*, *i* ≠ *M*+1. This implies that the hyperplane ℓ_0_(***x***) = 0, where

$$\begin{array}{l} \ell_{0}\left(\text{x}\right)=\langle \frac{Q\left(\text{x}\right)-\bar{Q}\left(\text{x}\right)}{R_{0}},\frac{Q\left({\text{x}}_{M+1}\right)-\bar{Q}\left(\text{x}\right)}{\left||Q\left({\text{x}}_{M+1}\right)-\bar{Q}\left(\text{x}\right)|\right|}\rangle -\frac{1}{\sqrt{2}} \end{array}$$

separates M and Y with probability *p* > 1−ϑ. □
